# Two-year cardio-pulmonary follow-up after severe COVID-19: a prospective study

**DOI:** 10.1007/s11739-023-03400-x

**Published:** 2023-09-16

**Authors:** Paola Faverio, Giuseppe Paciocco, Elena Tassistro, Paola Rebora, Emanuela Rossi, Anna Monzani, Marta Tundo, Chiara Milano, Martina Messa, Raffaele Marocchi, Alberto Pesci, Giuseppe Foti, Nicola Squillace, Viola Cogliandro, Maddalena Lettino, Maria Grazia Strepparava, Giuseppe Bellelli, Carlo Ferrarese, Maria Grazia Valsecchi, Paolo Bonfanti, Fabrizio Luppi, The STORM Long-COVID Team, Ester Pollastri, Ilaria Caramma, Anna Cappelletti, Luca Bonaffini, Laura Valagussa, Valerio Salvarani, Matteo Pozzi, Simone Beretta, Silvia Mori, Emanuela Rossi

**Affiliations:** 1 UOC Pneumologia, Respiratory Unit, Fondazione IRCCS San Gerardo dei Tintori, Via Pergolesi 33, 20900 Monza, Italy; 2 School of Medicine and Surgery, University of Milano Bicocca, Milan, Italy; 3 Bicocca Center of Bioinformatics, Biostatistics and Bioimaging, University of Milano Bicocca, Milan, Italy; 4 School of Medicine and Surgery, Anesthesia and Intensive Care Unit, University of Milano Bicocca, Fondazione IRCCS San Gerardo dei Tintori, Monza, Italy; 5 Infectious Disease Unit, Fondazione IRCCS San Gerardo dei Tintori, Monza, Italy; 6 Cardio-Thoracic and Vascular Department, Fondazione IRCCS San Gerardo dei Tintori, Monza, Italy; 7 Clinical Psychology Unit, School of Medicine and Surgery, University of Milano Bicocca, Monza, Italy; 8 School of Medicine and Surgery, Acute Geriatric Unit, University of Milano Bicocca, Fondazione IRCCS San Gerardo dei Tintori, Monza, Italy; 9 School of Medicine and Surgery, Neurology Unit, University of Milano Bicocca, Fondazione IRCCS San Gerardo dei Tintori, Monza, Italy

**Keywords:** COVID-19, Pneumonia, Pulmonary circulation, Pulmonary hypertension, Respiratory function tests

## Abstract

Short- and medium-term cardio-pulmonary sequelae after COVID-19 have been extensively studied. However, studies with longer follow-ups are required. This study aims to identify and characterise cardio-pulmonary sequelae, in patients hospitalised for SARS-CoV-2 pneumonia, at 24 months follow-up. This is a prospective, observational cohort study conducted on consecutive patients hospitalised for COVID-19 and acute respiratory failure. Patients were followed up at 24 months with complete pulmonary function tests (PFTs), 6-min walking test and a dyspnoea score (Modified Medical Research Council scale). A subgroup of patients with at least one clinical or functional sign suggestive of increased pulmonary pressures also underwent transthoracic echocardiography (TTE) to evaluate the presence of direct or indirect signs of pulmonary hypertension (PH). Ninety consecutive patients (74% men, median age 59.1 years) were enrolled in the study. In regard to PFTs, carbon monoxide diffusion capacity (DLCO) impairment was observed in 23 cases (26%), in all cases of mild entity. When considering the dyspnoea, 30 (34%) patients showed some degree of breathlessness. Forty patients underwent TTE. No patients had overt PH or chronic thromboembolic PH. However, all patients showed a hyperdynamic state of the right ventricle, and 8 (20%) patients had a decreased acceleration time on pulmonary valve, signs of increased pulmonary vasculature resistances and afterload elevation. At 24-month follow-up after severe COVID-19, DLCO and TTE prove to be the most sensitive tool to detect cardio-pulmonary sequelae. Dyspnoea is still present in about one-third of patients and requires a multidisciplinary approach.

**Supplementary Information:**

The online version contains supplementary material available at 10.1007/s11739-023-03400-x.

## Introduction

Short- and medium-term cardio-pulmonary sequelae after SARS-CoV-2 pneumonia have been extensively studied in the latest 2 years. Although fibrotic sequelae were reported in a minority of cases (36% at the 6-month and 26% at the 12-month follow-ups, in most cases of mild entity, according to a recent meta-analysis [1]), a higher proportion of patients still reported exertional dyspnoea and impaired carbon monoxide diffusion capacity (DLCO) at least until 1-year follow-up [2–4]. In regard to pulmonary vasculature involvement, the role of post-pulmonary embolism (PE) clinical syndrome has been less widely considered, regardless of a proven thrombotic event during SARS-CoV-2 pneumonia. Most studies describe a progressive cardio-pulmonary improvement between 6 and 12 months after COVID-19 infection [1, 4–8]. However, studies with longer (≥ 2 years) follow-up are required. Few studies available found a not-negligible proportion of patients (up to 20%) still reporting an incomplete recovery and persistence of symptoms, including dyspnoea and fatigue, at 24-month follow-up [9–11]. Furthermore, the long-term trajectory of lung function is still not clear, with some studies reporting a progressive improvement and others reporting a decline from 1 to 2 years after COVID-19 [11].

The severity of respiratory failure and the need of endotracheal intubation and invasive mechanical ventilation (IMV) during pneumonia were identified as some of the main factors associated to the development of both functional and radiological pulmonary sequelae [1, 4].

This study aims to identify and characterise cardio-pulmonary sequelae, in patients hospitalised for SARS-CoV-2 pneumonia, at 24-month follow-up, and to evaluate their association with the maximum ventilatory support received during hospitalisation.

## Materials and methods

This prospective, observational cohort study was performed in a 700-bed University hospital of northern Italy and enrolled consecutively adult patients hospitalised for laboratory-confirmed SARS-CoV-2 pneumonia and acute respiratory failure between March and June 2020. Patients with the following conditions were excluded: (1) New York Heart Association (NYHA) class IV heart failure; (2) pregnancy or breastfeeding; (3) severe renal failure (glomerular filtration rate lower than 30 ml/min); (4) bacterial and/or fungal pulmonary superinfection during hospital stay; (5) prior diagnosis of structural lung disease, including chronic obstructive pulmonary disease, emphysema, pulmonary fibrosis and/or bronchiectasis.

Patients were followed up at 6, 12 and 24 months from discharge to evaluate the presence of pulmonary sequelae. A clinical evaluation, including a dyspnoea score (Modified Medical Research Council (mMRC) scale), plethysmography and DLCO with single-breath technique and 6-min walking test (6MWT) was performed.

Data from 6- and 12-month follow-ups were already described in a previous multicenter study [1, 12]. Therefore, we focussed on the 24-month follow-up.

Patients were stratified according to the maximum oxygen/ventilatory support received during hospital stay: (1) oxygen therapy alone, including only patients with mild respiratory failure corrected with low-flow oxygen through nasal cannula; (2) continuous positive airway pressure (CPAP); (3) IMV.

The primary endpoint of the study was DLCO impairment (DLCO% < 80% of predicted) evaluated at 24 months from hospital discharge. The secondary endpoints of the study were also assessed at 24 months from hospital discharge, including: (1) Forced Vital Capacity (FVC), Tiffeneau Index (FEV1/FVC ratio), Forced Expiratory Volume in the 1st second (FEV1) and Total Lung Capacity (TLC) alterations; (2) variation from the expected of the normal distance walked on 6MWT; and (3) dyspnoea evaluated through mMRC scale.

A subgroup of patients with at least one of the following criteria, suggestive of pulmonary vascular function impairment, at the 24-month follow-up visit also underwent transthoracic echocardiography (TTE): (i) mMRC ≥ 1; (ii) desaturation at 6MWT ≥ 4%; (iii) FVC%/DLCO% ratio > 1.5; (iv) diagnosis of deep vein thrombosis and/or PE during the hospitalisation for SARS-CoV-2 infection. TTE studies were performed by the same operator (G.P.) with the same instrument (Esaote My lab Twice) following the most recent guidelines [13]. All patients were in sinus rhythm during TTE execution. TTE was performed at 24 months from hospitalisation and all the parameters described in the most recent Pulmonary Hypertension (PH) guidelines were measured to evaluate the presence of direct or indirect signs of PH [14, 15].

This study was approved by the Ethics Committee of Spallanzani Hospital (Clinical.Trial.gov Identifier: NCT04424992) and all patients signed informed consent.

Baseline characteristics were described as median (I and III quartiles, Q1–Q3) and frequencies (percentage). Differences between the three strata identified by the maximum ventilatory support received during hospital stay were compared by Fisher’s exact test or Kruskal–Wallis rank-sum test, as appropriate. Longitudinal transitions in mMRC values from 6-month through 12-month to 24-month follow-up are presented as an alluvial plot. The analyses were performed in R (version 4.0.4) and SAS (v 9.4).

## Results

Ninety consecutive patients (*N* = 67, 74% men; median [Q1–Q3] age 59.1 [53.7–63.4] years) were enrolled in the study and stratified as follows: 10 patients in the “oxygen alone” group, 46 patients in the “CPAP” group and 34 patients in the “IMV” group. The median [Q1–Q3] length of the hospitalisation for SARS-CoV-2 pneumonia in the oxygen only, CPAP and IMV group was 16 [12–19], 16 [8–22], 34 [26–39] days, respectively. The majority of patients were overweight and the most common comorbidities were cardiovascular diseases and hypertension, without differences between groups, Table 1. Patients in the IMV group were more intensively treated with systemic steroid, prophylactic heparin and remdesivir during the hospitalisation.

**Table 1:** Demographics and clinical characteristics of the 90 patients followed up at 24 months from COVID-19 hospitalisation

	Oxygen only (*N* = 10)	CPAP (*N* = 46)	IMV (*N* = 34)	*p*-value
Demographics				
Age (years), median (Q1–Q3)	59.1 (57.6–60.3)	56.9 (51.5–63.0)	60.1 (57.2–67.4)	0.023
Female gender, *N* (%)	7 (70)	10 (22)	6 (18)	0.005
Ex or never smokers, *N* (%)	9 (90)	44 (97)	31 (91)	0.077
BMI, median (Q1–Q3)	29.5 (28.0–30.9)	29.1 (26.8–31.0)	31.2 (27.4–34.7)	0.170
Comorbidities				
Cardiovascular diseases, *N* (%)	1 (10)	14 (30)	16 (47)	0.071
Hypertension, *N* (%)	2 (20)	5 (11)	9 (26)	0.196
Cerebrovascular diseases, *N* (%)	0 (0)	2 (4)	0 (0)	0.609
Asthma, *N* (%)	2 (20)	3 (7)	1 (3)	0.188
Diabetes, *N* (%)	0 (0)	1 (2)	5 (15)	0.094
No. of comorbidities, *N* (%)				
0	4 (40)	23 (50)	7 (21)	0.070
1	5 (50)	19 (41)	20 (59)	
≥ 2	1 (10)	4 (9)	7 (21)	
Treatments during hospitalisation for COVID-19				
Systemic steroid, *N* (%)	0 (0)	12 (26)	19 (56)	0.001
Prophylactic heparin, *N* (%)	7 (70)	20 (43)	24 (71)	0.037
Tocilizumab, *N* (%)	2 (20)	4 (9)	7 (21)	0.232
Remdesivir, *N* (%)	0 (0)	0 (0)	9 (26)	< 0.001
Mucolytics, *N* (%)	8 (80)	29 (63)	22 (65)	0.662
Pulmonary function tests				
FEV1%, median (Q1–Q3)	112.0 (107.2–120.8)	111.5 (98.0–116.8)	103.5 (98.0–116.0)	0.194
FEV1 (L), median (Q1–Q3)	3.1 (2.5–3.6)	3.5 (3.1–3.8)	3.0 (2.9–3.4)	0.075
FVC%, median (Q1–Q3)	107.0 (99.5–111.2)	106.0 (98.2–113.8)	100.0 (96.0–111.2)	0.417
FVC (L), median (Q1–Q3)	3.8 (3.1–4.3)	4.4 (3.8–5.0)	4.0 (3.4–4.4)	0.026
TI, median (Q1–Q3)	82.4 (80.1–83.4)	78.9 (75.5–82.7)	81.4 (77.4–83.1)	0.141
TLC%, median (Q1–Q3)	104.5 (101.2–115.0)	99.0 (94.0–106.8)	94.0 (89.5–99.5)	0.005
TLC (L), median (Q1–Q3)	5.2 (5.0–6.6)	6.3 (5.7–7.0)	5.9 (5.2–6.5)	0.064
DLCO%, median (Q1–Q3)	98.5 (88.8–104.0)	91.0 (82.2–96.5)	87.0 (75.2–94.8)	0.054
DLCO (mmoL/min*kPa), median (Q1–Q3)	7.0 [6.7, 9.9]	8.2 [6.7, 9.3]	7.1 [6.2, 8.5]	0.148
DLCO impairment, *N* (%)	1 (10)	10 (22)	12 (35)	0.232
Metres walked at 6MWT, median (Q1–Q3)	477.5 (471.2–518.8)	502.5 (435.0–538.8)	455.0 (425.0–483.8)	0.146
Distance walked lower than expected, *N* (%)	1 (10)	8 (18)	4 (12)	0.758
Oxygen saturation % at the beginning of the 6MWT	97.5 [97.0, 98.0]	96.5 [95.0, 97.0]	95.5 [94.0, 97.0]	0.002
Oxygen saturation % at the end of the 6MWT	99.0 [98.0, 99.0]	98.0 [97.0, 98.0]	98.0 [97.0, 98.8]	0.051
Dyspnoea scale				
mMRC scale, *N* (%)				
0	6 (60)	32 (71)	21 (62)	0.651
1	4 (40)	10 (22)	11 (32)	
2	0 (0)	3 (7)	1 (3)	
3	0 (0)	0 (0)	1 (3)	

*BMI* Body mass index, *FEV1* forced expiratory volume in the 1st second, *FVC* forced vital capacity; *TI* Tiffeneau index, *TLC* total lung capacity, *DLCO* carbon monoxide diffusion capacity, *6MWT* 6-min walking test, *mMRC* modified medical research council

During the 2-year follow-up, 9 patients (10%), with no differences between groups, were re-hospitalised mainly for surgical interventions (e.g. timectomy for timoma, thyroidectomy for multinodular struma and parathyroid adenoma removal), followed by one case of myocardial infarction and one patient with empyema due methicillin-resistant *S. aureus*. New diagnoses received after COVID-19 hospitalisation included mainly thyroid dysfunction (2 cases of thyroid nodules, 1 case of multinodular struma and 1 patient with Hashimoto’s thyroiditis), neurological disorders (1 case of Parkinson’s disease, 1 case of epilepsy, 1 case of hydrocephalus and 2 patients with episodes of confusion interpreted as post-traumatic stress disorder) and cardiac arrhythmias (2 cases of atrial fibrillation and 1 case of paroxysmal supraventricular tachycardia). Two patients out of 90 experienced a SARS-CoV-2 reinfection during the 24-month follow-up, in both cases of mild entity without need of rehospitalisation.

Regarding pulmonary function tests (PFTs) as continuous variables, median values at 24-month follow-up were normal for all parameters analysed, Table 1; however, TLC% and DLCO% were significantly lower in the IMV group compared to CPAP and oxygen only group. When considering abnormal values, only 3 patients (3%) showed a FVC% < 80% of predicted and 4 (4%) showed a TLC% < 80% of predicted, with no differences between groups. In all cases, the FVC and TLC impairment were mild. DLCO impairment (DLCO% < 80% of predicted) was observed in 23 cases (26%), more frequently in the IMV group although without statistical significance, and in all cases of mild entity (60–79% of predicted).

At 6MWT 13 patients (11%) showed a distance walked lower than expected, without differences between groups, and no patients required oxygen supplementation during the test. However, 5 cases showed a desaturation ≥ 4%.

When considering the degree of dyspnoea reported by patients through the mMRC scale, 30 (34%) showed some degree of breathlessness, with no differences between groups, Table 1.

Finally, in regard to pulmonary vasculature evaluation, 45 patients (50% of the entire cohort) met the criteria to perform the TTE, in particular: (i) 30 patients had a mMRC ≥ 1; (ii) 5 patients had a desaturation ≥ 4% at 6MWT; (iii) 7 patients had a FVC%/DLCO% ratio > 1.5; (iv) 7 patients received a diagnosis of deep vein thrombosis, and 3 of them also developed PE during hospitalisation for COVID-19. Since 5 patients refused to perform the TTE, 40 patients (68% men; median age 62 years) actually underwent the exam (18 (45%) in the IMV group, 14 (35%) in the CPAP group and 8 (20%) in the “oxygen only” group).

All TTE parameters described in the 2022 PH guidelines as early signs of PH were measured and reported in Table 2, with the exception of the peak systolic (S’) velocity of tricuspid annulus measured with tissue Doppler [14, 15]. None of the patients had overt PH. Only one case with marginally increased pulmonary artery systolic pressure (PASP) was suspected of having chronic thromboembolic PH (CTEPH), but the subsequent execution of a ventilation/perfusion lung scintigraphy ruled out this hypothesis. All 40 patients showed a hyper-dynamic state of the right ventricle with normal-to-elevated measures of right heart function and a tricuspid annular plane systolic excursion (TAPSE) of minimum 20 mm, whilst 8 (20%) patients had a decreased acceleration time (acT) on pulmonary valve [16], signs of increased afterload and pulmonary vasculature resistances. Five out of the 8 patients with a decreased acT also showed a desaturation ≥ 4% at 6MWT.

**Table 2:** Transthoracic eco-cardiographic parameters at 24-month follow-up

Parameters	*N* = 40
Left atrial area (cm^2^), median (Q1–Q3)	17.7 (15.6–21.0)
Right atrial area (cm^2^), median (Q1–Q3)	15.0 (13.0–16.3)
Ejection fraction %, median (Q1–Q3)	60 (55–60)
Right ventricular eccentricity index, median (Q1–Q3)	1 (1–1.1)
Acceleration time (acT) (msec), median (Q1–Q3)	120 (106–150)
acT < 100 ms, *N* (%)	8 (20)
Left ventricular end-diastolic diameter (mm), median (Q1–Q3)	49.6 (45.7–54.0)
Right ventricular end-diastolic diameter (mm), median (Q1–Q3)	39 (34–42)
Right atrium/left atrium ratio, median (Q1–Q3)	0.86 (0.75–0.96)
Right ventricle/left ventricle ratio, median (Q1–Q3)	0.78 (0.70–0.89)
Peak tricuspid regurgitation velocity (m/s), median (Q1–Q3)	2.0 (1.9–2.4)
Tricuspid pressure gradient (mm Hg), median (Q1–Q3)	17.5 (14–23)
Tricuspid annular plane systolic excursion (TAPSE) (mm), median (Q1–Q3)	25 (22–27)
TAPSE > 20 mm, *N* (%)	40 (100)
Derived Right Atrial Pressure (mm Hg), median (Q1–Q3)	5 (5–5)
Pulmonary artery systolic pressure (mm Hg), median (Q1–Q3)	25 (19–28.25)
Inferior vena cava diameter (mm), median (Q1–Q3)	11 (10–14)
Pericardial effusion, *N* (%)	0

## Discussion

To the best of our knowledge, this is the first prospective observational study that aimed to describe the long-term cardio-pulmonary sequelae after a severe COVID-19 infection.

In regard to involvement of lung parenchyma, PFTs were normal in most cases although DLCO abnormalities of mild entity were persistent in about one-fourth of patients at 24-month follow-up. Interestingly, patients requiring IMV during hospitalisation still showed a statistical significant reduction in both TLC and DLCO. However, this does not seem to be clinically meaningful since we did not observe a difference in the severity of dyspnoea or a reduction in the walked distance between patients who received IMV and the other groups.

DLCO has already been described as the most sensitive, appropriate non-invasive marker for evaluating pulmonary impairment at 6- and 12-month follow-ups [1, 4, 12], and the importance of this tool is confirmed at 24-month follow-up. Of note, DLCO impairment may be secondary to both lung parenchyma sequelae and pulmonary vascular function impairment.

Long-term pulmonary vasculature involvement after COVID-19 infection has not been yet systematically investigated. An increased risk of deep vein thrombosis up to three months and of PE up to six months after acute SARS-CoV-2 infection has been reported [17]. Whether such pulmonary vascular injury is transient and self-correcting or has the potential to lead to irreversible sequelae will only become apparent with longer-term follow-ups. In a recent study evaluating the onset of CTEPH during the follow-up of patients who developed acute PE during COVID-19 infection, 3 (23%) out of 18 cases developed CTEPH, diagnosed through scinti-single-photon emission computerised tomography and confirmed with right heart catheterization, in a 6-month period [18]. In our cohort, only a small proportion of patients received a diagnosis of PE during COVID-19 hospitalisation (3 out of 90 patients). Nevertheless, we performed a TTE measuring all the echocardiographic parameters predictor of PH development in a subgroup of 40 patients that, at 24-month follow-up, showed clinical and/or functional alterations suggestive of increased pulmonary pressures. Our study demonstrates that although 50% of patients in our cohort at 2-year follow-up still show some degree of clinical or functional alterations suggestive of impaired pulmonary vascular function, none of them had echocardiographic suspicion of CTEPH (low atrio-ventricular gradient). Although we did not find evidence of PH at TTE, we observed a decreased acT on pulmonary valve, parameter closely related to increased pulmonary vasculature resistances, in 20% of cases and a hyperdynamic right ventricle, which may be a marker of chronic afterload elevation before detectable PH [19], in all patients. Both these alterations may be considered early signs of chronic thromboembolic disease (CTED), a condition in which pulmonary emboli may have failed to resolve, but resting PH has not been developed, thanks to compensation mechanisms or the limited number of occluded segments of the vascular bed involved. We speculated that these two findings may be the consequence of an anatomical obstruction of pulmonary vasculature secondary to in situ microthrombosis widely described in the acute phase of severe COVID-19 [20]. Prior post-mortem studies including the histopathologic data of deceased patients with SARS-CoV-2 pneumonia showed widespread pulmonary microthrombosis secondary to disease-specific hypercoagulability [20]. Furthermore, our cohort of patients suffered SARS-CoV-2 infection during the first pandemic wave (February–May 2020). At that time, prophylactic heparin was not yet standard of care in severe COVID-19 and only 57% of our cohort received such treatment. This may have favoured the hypercoagulable state and the formation of microthrombi.

Finally, this analysis follows a prior study describing the 12-month follow-up after SARS-CoV-2 infection [1], confirming a slow but progressive improvement of PFTs between 6- and 12-month follow-ups: 45.5% vs 40% DLCO% impairment at 6 months vs. 1 year and 16.7% vs 11.5% TLC% impairment at 6 months and 1 year, respectively. The analysis on the subgroup of patients followed up at 2 years seems to confirm this trend, with 26% and 4% of patients still showing DLCO% and TLC% impairment, respectively.

**Fig. 1:**
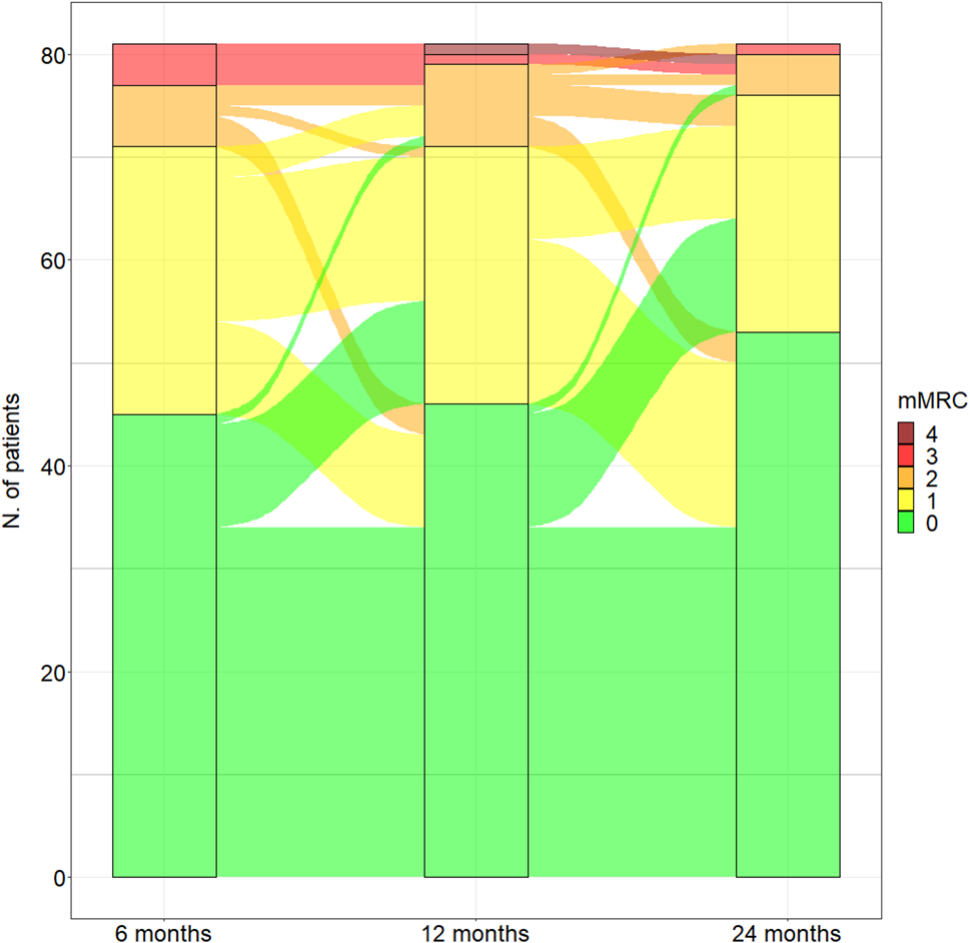
Alluvial plot describing the trend of dyspnoea evaluated through mMRC scale at 6-month, 12-month and 24-month follow-ups. *mMRC* modified medical research council. *N* = 81 patients who had mMRC evaluation at the three follow-up times

Of note, examining only the 81 patients with mMRC values available at all 3 follow-up time-points (6-month, 12-month and 24-month) dyspnoea showed a slower trend of improvement compared to PFTs (44% patients at 6 months *vs* 43% at 1 year and 35% at 2 years showed a mMRC ≥ 1) and a certain degree of variability between visits, as shown in Fig. 1.

The mechanisms underlying shortness of breath in patients showing persisting symptoms 2 years after COVID-19 hospitalisation could be not simply related to cardio-pulmonary impairment but can be related to the so-called “long-COVID” syndrome [21], which includes systemic symptoms as well, such as fatigue and cognitive dysfunction. Multicomponent exercise programmes including intervallic training sessions followed by strengthening exercises and individualised respiratory physiotherapy have proven to be effective in reducing the disability due to dyspnoea at 2-year follow-up after acute COVID-19 [22]. Furthermore, one-third of patients in our cohort required IMV and intensive care unit admission during the hospitalisation for SARS-CoV-2 pneumonia. Therefore, in this subset of cases, also the post-intensive care syndrome may have played a role on long-term sequelae [23]. Such findings suggest that the persistence of dyspnoea after 2 years from SARS-CoV-2 infection requires an accurate cardio-pulmonary diagnostic work-up. Nevertheless, a multidisciplinary approach including also physiotherapists and psychologists may help in the differential diagnosis on long-COVID syndrome.

Our study presents some limitations. First, the small sample size. Second, we did not perform systematic TTE in all patients in our cohort. Third, our study included consecutive patients from the first pandemic wave recruited in a single tertiary-care hospital in northern Italy, and this may limit the generalizability of the results.

Future studies should aim at including systematic long-term evaluation of pulmonary vasculature function in the follow-up of patients with severe COVID-19. Furthermore, performing long-term cardio-pulmonary follow-ups in patients who suffered SARS-CoV-2 infection during more recent pandemic waves during which PE was more likely to be suspected and diagnosed and prophylactic heparin had become standard of care may help to better evaluate the role of pulmonary microthrombosis.

In conclusion, the trajectory of long-term cardio-pulmonary sequelae after SARS-CoV-2 pneumonia is not yet completely understood. At 24-month follow-up, DLCO is confirmed to be the most sensitive tool with 26% of patients that still show mild alteration and a higher degree of impairment in those receiving IMV during hospitalisation. Fifty per cent of our cohort showed clinical and/or functional alterations suggestive of increased pulmonary pressures and underwent to TTE. No patients had overt PH or CTEPH; however, all of them had early signs of CTED, including a hyperdynamic state of the right ventricle and a decrease of acT on pulmonary valve. Exertional dyspnoea is still present in 34% of patients with a slow but progressive improvement compared to 6-month and 12-month follow-ups. In patients with persisting dyspnoea at long-term follow-up after COVID-19, multidisciplinary diagnostic work-up should be routinely performed.

### Supplementary Information

Below is the link to the electronic supplementary material.

Supplementary file1 (XLSX 33 KB)

## Data Availability

Individual participant data referring to this article (i.e. text, tables and figures) will be made available upon reasonable request. Proposals should be directed to paola.faverio@unimib.it
